# Treatment of myofascial trigger points in common shoulder disorders by physical therapy: A randomized controlled trial [ISRCTN75722066]

**DOI:** 10.1186/1471-2474-8-107

**Published:** 2007-11-05

**Authors:** Carel Bron, Michel Wensing, Jo LM Franssen, Rob AB Oostendorp

**Affiliations:** 1 Research Centre for Allied Health Sciences, Centre for Quality of Care Research, Radboud University Medical Centre, Nijmegen, The Netherlands; 2 Centre for Quality of Care Research, Radboud University Medical Centre, Nijmegen, The Netherlands; 3 Private Practice for Physical therapy for Neck, Shoulder and Upper Extremity Disorders, Groningen, The Netherlands; 4 Dutch Institute for Allied Health Care, Amersfoort, The Netherlands

## Abstract

**Background:**

Shoulder disorders are a common health problem in western societies. Several treatment protocols have been developed for the clinical management of persons with shoulder pain. However available evidence does not support any protocol as being superior over others. Systematic reviews provide some evidence that certain physical therapy interventions (i.e. supervised exercises and mobilisation) are effective in particular shoulder disorders (i.e. rotator cuff disorders, mixed shoulder disorders and adhesive capsulitis), but there is an ongoing need for high quality trials of physical therapy interventions. Usually, physical therapy consists of active exercises intended to strengthen the shoulder muscles as stabilizers of the glenohumeral joint or perform mobilisations to improve restricted mobility of the glenohumeral or adjacent joints (shoulder girdle). It is generally accepted that a-traumatic shoulder problems are the result of impingement of the subacromial structures, such as the bursa or rotator cuff tendons. Myofascial trigger points (MTrPs) in shoulder muscles may also lead to a complex of symptoms that are often seen in patients diagnosed with subacromial impingement or rotator cuff tendinopathy. Little is known about the treatment of MTrPs in patients with shoulder disorders.

The primary aim of this study is to investigate whether physical therapy modalities to inactivate MTrPs can reduce symptoms and improve shoulder function in daily activities in a population of chronic a-traumatic shoulder patients when compared to a wait-and-see strategy. In addition we investigate the recurrence rate during a one-year-follow-up period.

**Methods/Design:**

This paper presents the design for a randomized controlled trial to be conducted between September 2007 – September 2008, evaluating the effectiveness of a physical therapy treatment for non-traumatic shoulder complaints. One hundred subjects are included in this study. All subjects have unilateral shoulder pain for at least six months and are referred to a physical therapy practice specialized in musculoskeletal disorders of the neck-, shoulder-, and arm.

After the initial assessment patients are randomly assigned to either an intervention group or a control-group (wait and see). The primary outcome measure is the overall score of the Dutch language version of the DASH (Disabilities of Arm, Shoulder and Hand) questionnaire.

**Discussion:**

Since there is only little evidence for the efficacy of physical therapy interventions in certain shoulder disorders, there is a need for further research. We found only a few studies examining the efficacy of MTrP therapy for shoulder disorders. Therefore we will perform a randomised clinical trial of the effect of physical therapy interventions aimed to inactivate MTrPs, on pain and impairment in shoulder function in a population of chronic a-traumatic shoulder patients. We opted for an intervention strategy that best reflects daily practice. Manual high velocity thrust techniques and dry-needling are excluded. Because in most physical therapy interventions, blinding of the patient and the therapist is not possible, we will perform a randomised, controlled and observer-blinded study.

**Trial Registration:**

This randomized clinical trial is registered at current controlled trials ISRCTN75722066.

## Background

Shoulder pain is a common health problem in western societies. There are substantial differences in reported prevalence in the general population. The one-year prevalence of shoulder disorders has been reported to range from 20% to 50%. This wide range is strongly influenced for example by the definition of shoulder disorders, including or excluding limited motion, age, gender and anatomic area [1-3]. Of all shoulder patients who attend primary care physicians 50% recover within 6 months, meaning they do not seek any medical help after the first episode[1,4-6]. Chronicity and recurrence of symptoms are common [7,8]. According to the guidelines of the Dutch College of General Practioners [9], the recommended management of shoulder symptoms starts with educational information about the natural course of shoulder pain combined with the advise to avoid irritating and loading activities. The use of analgesics or NSAIDs is recommended for the first two weeks. When no recovery occurs within two weeks, subacromial or intra-articular injection therapy with corticosteroids are administered and eventually repeated. Finally, physical therapy is only recommended after a 6-week period when there are functional limitations (specifically an activating and time-contingent approach). International guidelines for shoulder pain, including the Clinical Guideline of Shoulder pain of the American Academy of Orthopaedic Surgeons [10] and the Shoulder Guideline of the New Zealand Guidelines Group[11] differ more or less from the Dutch guidelines in classification, recommended interventions and timeline, and order of interventions. Scientific evidence from randomized clinical trials, meta-analyses or systematic reviews for either the efficacy of multimodal rehabilitation, injection therapy, medication, surgery or physical therapy or the order of application of commonly used therapies is lacking [12-16].

An alternative approach to the management of persons with shoulder problems consists of a treatment aimed at inactivating MTrPs and eliminating factors that perpetuate them. MTrPs may be inactivated by manual techniques (such as compression on the trigger point or other massage techniques), cooling the skin with ethyl chloride spray or with ice-cubes followed by stretching of the involved muscle, trigger point needling using an acupuncture needle, or injection with local anaesthetics or Botulinum toxin, followed by ergonomic advises, active exercises, postural correction, and relaxation (with or without biofeedback)[17,18]. Over the years, MTrPs are increasingly accepted in the medical literature. Clinical, histological, biochemical and electrophysiological research has provided biological plausibility for the existence of MTrPs [19-24].

MTrPs are defined as exquisitely tender spots in discrete taut bands of hardened muscle that produce symptoms [25,26]. A previous study showed that MTrPs can be detected reliably by trained physiotherapists [27]. Palpation is still the only reliable method to diagnose myofascial pain clinically. In reviews addressing the efficacy of interventions in shoulder patients, MTrP therapy and myofascial pain are rarely mentioned [15]. However, some published case studies suggest that treatment of MTrPs in shoulder patients may be beneficial [28-31].

The primary aim of this study is to investigate the effectiveness of inactivation of MTrPs in shoulder muscles by physical therapy on symptoms and functioning of the shoulder in daily activities in a population of chronic a-traumatic shoulder patients when compared to a wait-and-see strategy. In addition, we investigate the recurrence rate during a one-year-follow-up period.

## Methods/Design

An examiner-blinded randomized controlled trial will be conducted, which has been approved by the ethics committee of the Radboud University Nijmegen Medical Centre, the Netherlands [CMO 2007/022].

### Participants/Study sample

Between September 2007 and September 2008, all consecutive patients referred to a physical therapy practice specialized in the treatment of individuals with musculoskeletal disorders of the neck, shoulder and arm are potential study participants. The referring physicians include general practioners, orthopaedic surgeons, neurologists and physiatrists. Patients are eligible if they have unilateral shoulder complaints (described as pain felt in the shoulder or upper arm) for at least six months. The patients present with persistent shoulder pain that has not spontaneously recovered. The patients are between 18 and 65 years old. Because the questionnaires are in the Dutch language, subjects must understand written and verbal Dutch. Patients who have been diagnosed (prior to the referral) with shoulder instability, shoulder fractures, systemic diseases (such as rheumatoid arthritis, Reiter's syndrome, diabetes), or who's medical history or examination suggests neurological diseases, or other severe medical or psychiatric disorders will be excluded from the study. The project leader will check all the available information from referral letters, additional information from the general practitioner and from the patients. All eligible patients will be informed of the study and will be invited to participate. Patients who are willing to participate will be asked to review and sign the written informed consent.

### Measurements

**Figure 1:**
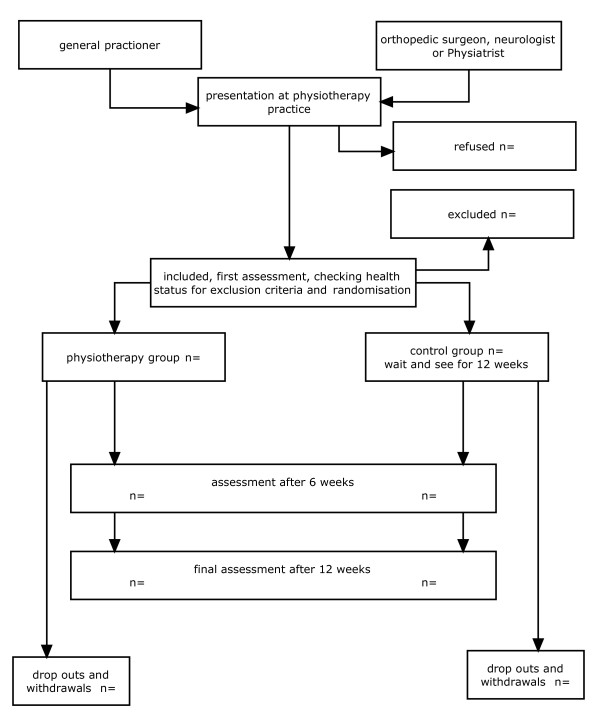
recruitment and experimental plan.

Before randomization, all participants will be assessed during an individual baseline test session. They will complete a battery of questionnaires and tests, determining data on social, demographic, and physical factors, and baseline values for the outcome measures. In addition, subjects will complete the DASH, RAND-36-dutch language version, and passive range of motion tests of the shoulder (PROM). During the initial assessment, MTrPs will be identified, based on compression-produced pain that is recognized by patients as their own shoulder pain. If no MTrPs are detected, the subjects will be excluded from the study. All measurements will be performed by the same independent observer, who is not employed by the physical therapy practice (This is to create optimal blinding of the observer, who is now not able to recognise the subjects). The observer is trained in identifying MTrPs and has several years of clinical experience in MTrP therapy. The observer participated in a former reliability study of MTrP palpation. The baseline measurements will be at T0, the second measurement (T1) will be 6 weeks after the first assessment session, the third (T2) will be 12 weeks after the first assessment session. All measurements [see box 1] will be performed outside the physical therapy practice to assure that the observer will not recognise any of the study participants when they come to the physical therapy practice for their treatment. After this first assessment, the patients will be randomly assigned to one of two groups: the intervention group or the control group. The patients in the control group will stay on the waiting list and will not receive any treatment. They are allowed to use over-the-counter painkillers during this 12-week period. After 6 weeks and 12 weeks, respectively, they will be examined by the same blinded observer. After 12 weeks they will receive the same physical therapy program as the experimental group [see Figure 1]. The initial trial ends after 12 weeks, but 6 months and 12 months after the start of the experimental intervention shoulder function of the subjects will be re-evaluated with the DASH-Dutch language version.

### Intervention

The patients in the intervention group will be treated by a physical therapist once a week for a maximum period of 12 weeks. All participating physiotherapists are experienced in treating patients with long-lasting shoulder symptoms and patients with MTrPs and myofascial pain, especially in the upper part of the body. They are trained and skilled in the identification of MTrPs and received a certification in manual trigger point therapy. The treatment starts with inactivation of the active (pain producing) MTrPs by using manual techniques (compression on the trigger point, manual stretching of the trigger point area and the taut band) combined with "intermittent cold application by using ice-cubes followed by stretching the muscle" according to Travell [32] to further inactivate the MTrPs. Manual pressure will decrease the sensitivity of the painful nodule in the muscle while other massage technique will mobilize and stretch the contracted muscle fibres. The application of the ice-cubes has a desensitizing effect, and makes it easier to stretch shoulder muscles. Each treatment session will end with a heat application to increase the circulation of the involved muscles.

Patients will be advised to do stretching exercises and will be taught to perform these correctly by means of surface-electromyography-assisted stretching[33,34]. Furthermore they will be advised to perform relaxation exercises, and to applyheat (like a hot shower, hot packs) several times (at least twice) a day. If there is abnormal measurable higher electromyographic activity in the upper trapezius muscle (measured by surface Electromyography (sEMG) using a Myomed 932 [Enraf Nonius, Delft, the Netherlands]) during standing and/or sitting [35], relaxation exercises will be performed using a portable myofeedback device (Myotrac I, Thought Technology, Quebec, Canada). Abnormal sEMG activity is defined as a constantly measured value above 1%–5% of the maximally voluntary contraction [36-39], which is in general above 10 microvolt, during several minutes and the patient is not able to relax the muscle spontaneously or on request. Finally, all patients will receive ergonomic recommendations, andinstructions to assume and maintain "good" posture [40,41]. Manual high velocity thrust techniques of the cervical spine and the shoulder and dry needling are excluded from the treatment protocol, because not all participating physical therapists are skilled to perform these techniques. The content of each session may vary as it depends on the findings during the first treatment session and the results of the previous treatment sessions. Thus, there are differences in the content of the individual treatments, but within the limits of the treatment protocol.

### Stoprule

The treatment ceases when the patient is completely symptom-free or the patient and the therapist agree that treatment will not further benefit the patient, although their participation in the study will prolong. If patients decide that they no longer wish to participate in the study they are free to withdraw from the study at any moment.

### Control of intervention integrity

To enhance the integrity of this complex intervention, every week all participating physical therapists will discuss the content of each therapy session with the researcher (CB) without mentioning names or other information which will assure the blinding of the independent researcher (CB). After 6 and 12 weeks, the patients of the intervention group will interviewed about the content of the received treatment sessions to assure that all patients will be treated according to the protocol. If patients are not treated according to the protocol, they will be identified and participation may be discontinued.

### Expectations regarding treatment outcome

At the start of the trial (T0) both the patients and physical therapists will complete a questionnaire regarding the anticipated treatment outcome.

### Setting

The study will be conducted in a physical therapy practice specialized in management of persons with musculoskeletal disorders of the neck, shoulder and arm. After randomisation every patient assigned to the experimental group will be treated by the same physical therapist.

### Objectives

In the current study we will test the following hypothesis (H0).

A physical therapy treatment to inactivate MTrPs within a three months' period is as effective as a "wait and see" approach of patients with chronic shoulder complaints in a three month period.

### Population characteristics

• To identify potential confounding factors, demographic information for all subjects will be collected including age, gender, education, occupation, sports and leisure activities, duration of the complaints, and type of onset, among others.

• The Dutch language version of the RAND-36 item Health Survey will be used for base line characteristics of the study population. The RAND-36, which is almost identical to the MOS SF-36 [42], scores the functional status and quality of life and is widely used for screening health status in medical, social and epidemiological research. The RAND-36 consists of 36 items divided into 8 subscales concerning physical functioning, role limitations due to physical health, role limitations due to emotional problems, energy and fatigue, emotional well-being, social functioning, pain, general health perception and health change. This questionnaire is considered to be a reliable instrument for comparing groups (internal consistency Cronbach's alpha > 0.70). The test-retest stability is sufficient (0.58 – 0.82) and the questionnaire is responsive when scoring after at least 4 weeks. The construct validity was estimated by comparing the RAND-36 with other Health questionnaires (like the Nottingham Health Profile [NHP] and the Groninger Activities Restriction Scale [GARS]. There are significant correlations between the subscales of the RAND-36 and the subscales of the NHP (correlation coefficient 0.42 – 0.69). The correlation coefficient between the subscale physical functioning and the GARS is 0.65. A higher score (maximum is 100 points) defines a more favourable health status.

• The Beck Depression Inventory (BDI) is used to discriminate between patients with major depression and those without or with minor depressive feelings. The BDI is included because depression may be a confounding factor. The BDI is widely accepted and used in clinical and experimental research and its predictive value is rated as good. A BDI-score equally or higher than 21 indicates a major depression (specificity 78.4%) [43].

### Outcomes

The following outcome parameters will be used:

#### Primary

The overall score of the DASH (Disability of Arm Shoulder and Hand) questionnaire – Dutch language version will be used as the primary outcome measure. The DASH is a multidimensional (physical, emotional and social) 30-item self-report measure focussing on physical function pain and other symptoms. At least 27 of the 30 items must be completed for a score to be calculated. The assigned values for all completed responses are simply summed and averaged. This value is then transformed to a score out of 100 by subtracting one and multiplying by 25. The transformation is done to make the score easier to compare to other measures using a 0–100 scale. A higher score indicates greater disability.

$$\text{DASH disability/sympton score}=\frac{\left[(\text{sum of n responses})-1\right]}{\text{n}}\times 25$$

where n is equal to the number of completed responses.

Scoring is on a 5-point Likert scale from no difficulty (0 points) to very difficult (5 points). The range of the total score is from 0 to 100, where 0 means no symptoms (pain, tingling, weakness or stiffness) and no difficulty in performing daily activities, while 100 means extreme, severe symptoms and unable to perform any daily activity. Content and face validity of the DASH were confirmed by a variety of experts of the American Academy of Orthopaedic Surgeons (AAOS), the council of Musculoskeletal Speciality Societies (COMSS) and the institute for Work and Health (Toronto, Ontario, Canada) throughout the development process [44].

Its internal consistency was excellent (Cronbach's alpha = 0.96) during field-testing. The test-retest reliability was excellent (ICC_2.1 _= 0.92 and 0.96) in two studies [45,46] and satisfactory in one study (Pearson 0.98 and kappa 0.67). The minimal detectable Change (MDC) was calculated in a population of 172 patients with several upper limb disorders (Osteoarthritis, Carpal Tunnel syndrome, Rotator Cuff syndrome, Rheumatoid Arthritis and Tennis Elbow) [47]. The Minimal Detectable Change (MDC) varied between 10.70 (at 90% confidence level) and 12.75 (at 95% confidence level). The DASH demonstrated to be a responsive questionnaire.

The inter- and intra-observer reliability is good to excellent (intra-observer reliability Pearson r = 0.96 to 0.98; ICC = 0.91 to 0.96; Inter-observer agreement Cohen's kappa = 0.79).

The construct validity was estimated by comparing the DASH to several other questionnaires. The correlation with other instruments like the SPADI (Shoulder Pain and Disability Index) is good (Pearson's r = 0.82 to 0.88). The DASH questionnaire is one of the best among 16 other questionnaires for shoulder symptoms [48].

#### Secondary

An independent examiner will perform the following tests.

• The total number of shoulder muscles with MTrPs will be counted and compared to the baseline measurement findings.

• Passive range of motion of the shoulder will be measured by an handheld digital inclinometer (The Saunders group Inc, Chaska, MN). The range of motion of the non-painful shoulder will be used as reference [49,49,50]. Because the normal range of motion differs from one individual to another, we focus on improvement of limited range of motion during the experiment (both experimental group and control group).

◦ For the measurement of passive external rotation, the patient is in a supine position, with the shoulder in 0° of abduction and rotation, the elbow flexed at 90° and the forearm in a neutral position. This position is defined as the position of 0°. The observer then performs external rotation until pain limits the range of motion or the extreme of the range is reached. The inclinometer is placed against the volar side of the forearm. This range of motion is recorded in degrees. The normal range of motion for external rotation is between 70° and 90°.

◦ For the measurement of passive glenohumeral abduction, the patient is seated upright, and the position of 0° is defined as the upper arm is in a neutral position. While palpating the lower angle of the scapula with the thumb, the examiner elevates the upper arm of the patient until the scapula begins to rotate or pain limits further motion. The inclinometer is placed against the lateral side of the upper arm near the elbow. The range of motion is recorded in degrees. The normal range of motion is 90°.

◦ For the measurement of passive elevation (through flexion), the patient is in the supine position with the arm along the side. This position is defined as the position of 0°. The observer than performs elevation until pain limits the range of motion or the extreme of the range is reached. Then the inclinometer is placed against the medial side of the upper arm near the elbow. The range of motion is recorded in degrees. The normal range of motion is between 165° and 180°

◦ For the measurement of internal rotation the patient is in a prone position. The shoulder is 90° abduction, and the forearm is in neutral position. This position is defined as the position of 0°. The observer than performs internal rotation until pain limits the range of motion or the extreme of the range is reached. The sensor is placed against the volar side of the forearm. The normal range of motion is 70°

◦ For the measurement of horizontal adduction the patient is in a supine position. The arm is in 90° abduction. This position is defined as the position of 0°. The observer performs adduction, while the arm stays in the vertical plane, until pain limits the range of motion or the extreme of the range is reached. The normal range of motion is 135°

• Finally the total number of treatment sessions will be counted. This is done by an assistant, who is not involved in the study by using the administration-software of the practice [see Table 1].

**Table 1:** Overview of variables

**Variable**	**T0 Baseline**	**T1 After 6 wk**	**T2 After 12 wk**	**Measured by**
Age*	X			Interview
Gender*	X			Interview
***Work***	X			Interview
***Dominant side affected***	X			Interview
Duration of the complaints*	X			Interview
***DASH ***DLV	X	X	X	Questionnaire
Use of ***medication***	X	X	X	Interview
Use of ***other therapy***	X	X	X	Interview
***Work ***%	X	X	X	Interview
***Improvement ***(percentage of perceived improvement)		X	X	Interview
***Number of involved muscles***	X	X	X	Assessment
***No. of treatment sessions***			X	Assessment
***Health status ***for baseline comparison	X			RAND-36 DLV
***Existence and severity of symptoms of depression***	X			Beck Depression Inventory
***Shoulder Passive ROM***	X	X	X	Goniometry
• flexion	X	X	X	
• abduction	X	X	X	
• external rotation	X	X	X	
• internal rotation	X	X	X	
• cross body adduction	X	X	X	

*Age, gender and duration of the complaints seem to be important prognostic variables [53].

### Sample size

The initial sample size is based on the assumption that the overall score of the primary outcome measure DASH shows a mean improvement of 15 points [SD = 22] [51]. To test the null hypothesis of equality of treatment at α = .05 with 90% power and assuming a uniform dropout rate of 5%, it was calculated that 52 patients in each group would be sufficient.

### Randomization

After inclusion the patients will be randomly assigned to either the intervention group or the "wait and see" group. The randomisation will be performed by an assistant not otherwise involved in the study by generating random numbers using computer software. Stratification or blocking strategies will not be used.

### Informed consent

The patients will be informed about the study prior to the first assessment and will be asked to give written informed consent.

### Blinding

Blinding of the patients or the physical therapists, who are involved in the treatment, is impossible due to the treatment characteristics.

An independent observer will collect baseline data and outcome data. The independent observer is blinded. The successfulness of the blinding procedure will be evaluated by asking the observer to which group she believes the subjects belong.

### Statistical analysis

For comparisons of prognostic variables on baseline we will use the Student's t test for continuous variables with normal distribution and the chi-square test for categorical variables or continuous variables with non-normal distribution[52]. For the overall score of the DASH (primary outcome measure) we will use the unpaired t-test for normally distributed data or Mann-Whitney Rank Sum-test for non-normally distributed data to assess the difference between the two groups after the treatments. Regression analyses will be used to include prognostic factors, such as the baseline scores like age, gender and duration of the complaints, in the analyses. All significance levels will be set at p < 0.05. All data will be analysed primarily according to intention-to-treat principle. We will use Sigmastat 3.11 and Systat 12 for windows (Systat Inc. Richmond, California, USA) for the statistical analyses.

## Discussion

Since there is little evidence for the efficacy of physical therapy interventions in some shoulder disorders, there is a need for further research. Therefore we will perform a randomised clinical trial dealing with the effect of physical therapy interventions aimed to inactivate MTrPs on pain and impairment in shoulder function in a population of chronic a-traumatic shoulder patients. To the best of our knowledge, few studies of the efficacy of MTrP therapy are published. We choose for an intervention strategy that best reflects daily practice. We excluded manual high velocity thrust techniques and intramuscular MTrP release by dry needling, because these interventions are not commonly used by Dutch physical therapists and not all participating therapists were skilled to perform these techniques at the beginning of the study. In most physical therapy interventions, blinding of the patient and the therapist is not possible. The observers will be blinded for the allocation procedure. The results of this trial will be presented as soon as they are available.

## Competing interests

The author(s) declare that they have no competing interests.

## Authors' contributions

All authors read, edited and approved the final manuscript. CB is the lead investigator, and developed the design of the study, will carry out data-acquisition, analysis, interpretations, and prepared as primary author the manuscript. MW and RO were responsible for the design, project supervision and writing of the manuscript. JF will assist in carrying out data acquisition and was involved in preparing the study design and in writing the manuscript.

## Pre-publication history

The pre-publication history for this paper can be accessed here:



## References

[B1] Luime JJ, Koes BW, Hendriksen IJ, Burdorf A, Verhagen AP, Miedema HS, Verhaar JA (2004). Prevalence and incidence of shoulder pain in the general population; a systematic review. Scand J Rheumatol.

[B2] Bongers PM (2001). The cost of shoulder pain at work. BMJ.

[B3] Pope DP, Croft PR, Pritchard CM, Silman AJ (1997). Prevalence of shoulder pain in the community: the influence of case definition. Ann Rheum Dis.

[B4] Bergman GJD (2005). Manipulative therapy for shoulder complaints in general practice.

[B5] van der Windt DA, Koes BW, Boeke AJ, Deville W, de Jong BA, Bouter LM (1996). Shoulder disorders in general practice: prognostic indicators of outcome. Br J Gen Pract.

[B6] HSJ P, van GHWV, JSAG S (2000). Klachten van het bewegingsapparaat in de Nederlandse bevolking. Prevalenties, consequenties en risicogroepen..

[B7] Mitchell C, Adebajo A, Hay E, Carr A (2005). Shoulder pain: diagnosis and management in primary care. BMJ.

[B8] Winters JC, Jorritsma W, Groenier KH, Sobel JS, Jong BM, Arendzen HJ (1999). Treatment of shoulder complaints in general practice: long term results of a randomised, single blind study comparing physiotherapy, manipulation, and corticosteroid injection. BMJ.

[B9] Winters JC, Jongh AC, van der Windt DAWM (1999). NHG standaard schouderklachten [Guidelines for Shoulder Complaints of the Dutch College of General Practioners (version 1999).. Huisarts Wet.

[B10] (2001). Clinical Guideline of Shoulder pain of the American Academy of Orthopaedic Surgeons.

[B11] (2004). Shoulder Guideline of the New Zealand Guidelines Group.

[B12] Karjalainen K, Malmivaara A, van TM, Roine R, Jauhiainen M, Hurri H, Koes B (2001). Multidisciplinary biopsychosocial rehabilitation for neck and shoulder pain among working age adults: a systematic review within the framework of the Cochrane Collaboration Back Review Group. Spine.

[B13] Green S, Buchbinder R, Hetrick S (2003). Physiotherapy interventions for shoulder pain. Cochrane Database Syst Rev.

[B14] Green S, Buchbinder R, Glazier R, Forbes A (2000). Interventions for shoulder pain. Cochrane Database Syst Rev.

[B15] Green S, Buchbinder R, Hetrick S (2005). Acupuncture for shoulder pain. Cochrane Database Syst Rev.

[B16] Ejnisman B, Andreoli CV, Soares BG, Fallopa F, Peccin MS, Abdalla RJ, Cohen M (2004). Interventions for tears of the rotator cuff in adults. Cochrane Database Syst Rev.

[B17] Simons DG, Travell JG, Simons LS, Travell JG (1999). Travell & Simons' myofascial pain and dysfunction The trigger point manual.

[B18] Baldry P (2004). Acupuncture, Trigger Points and Musculoskeletal Pain.

[B19] Gerwin RD, Dommerholt J, Shah JP (2004). An expansion of Simons' integrated hypothesis of trigger point formation. Curr Pain Headache Rep.

[B20] Hong CZ, Simons DG (1998). Pathophysiologic and electrophysiologic mechanisms of myofascial trigger points. Arch Phys Med Rehabil.

[B21] Mense S, Simons DG, Hoheisel U, Quenzer B (2003). Lesions of rat skeletal muscle after local block of acetylcholinesterase and neuromuscular stimulation. J Appl Physiol.

[B22] Shah JP, Phillips TM, Danoff JV, Gerber LH (2005). An in vivo microanalytical technique for measuring the local biochemical milieu of human skeletal muscle. J Appl Physiol.

[B23] Simons DG, Hong CZ, Simons LS (2002). Endplate potentials are common to midfiber myofacial trigger points. Am J Phys Med Rehabil.

[B24] Dommerholt J, Bron C, Franssen JLM (2006). Myofascial trigger points; an evidence-based review. J Manual Manipulative Therapy.

[B25] Wolfe F, Simons DG, Fricton J, Bennett RM, Goldenberg DL, Gerwin R, Hathaway D, McCain GA, Russell IJ, Sanders HO, . (1992). The fibromyalgia and myofascial pain syndromes: a preliminary study of tender points and trigger points in persons with fibromyalgia, myofascial pain syndrome and no disease. J Rheumatol.

[B26] Gerwin RD, Shannon S, Hong CZ, Hubbard D, Gevirtz R (1997). Interrater reliability in myofascial trigger point examination. Pain.

[B27] Bron C, Wensing M, Franssen JLM, RAB O (2007). Interobserver Reliability of Palpation of Myofascial Trigger Points in Shoulder Muscles. Journal Manual Manipulative Therapy.

[B28] Ingber RS (2000). Shoulder impingement in tennis/racquetball players treated with subscapularis myofascial treatments. Arch Phys Med Rehabil.

[B29] Weed ND (1983). When shoulder pain isn't bursitis. The myofascial pain syndrome. Postgrad Med.

[B30] Grosshandler SL, Stratas NE, Toomey TC, Gray WF (1985). Chronic neck and shoulder pain. Focusing on myofascial origins. Postgrad Med.

[B31] Bron C, Franssen JLM, de Valk BGM (2001). Een posttraumatische schouderklacht zonder aanwijsbaar letsel. Ned Tijdschrift v Fysiotherapie.

[B32] JG T, DG S (1999). Myofascial Pain and Dysfunction The Trigger Point Manual The lower extremities.

[B33] Neblett R, Gatchel RJ, Mayer TG (2003). A clinical guide to surface-EMG-assisted stretching as an adjunct to chronic musculoskeletal pain rehabilitation. Appl Psychophysiol Biofeedback.

[B34] Neblett R, Mayer TG, Gatchel RJ (2003). Theory and rationale for surface EMG-assisted stretching as an adjunct to chronic musculoskeletal pain rehabilitation. Appl Psychophysiol Biofeedback.

[B35] Franssen JLM, Franssen JLM (1995). Handboek oppervlakte-elektromyografie.

[B36] Veiersted KB, Westgaard RH, Andersen P (1990). Pattern of muscle activity during stereotyped work and its relation to muscle pain. Int Arch Occup Environ Health.

[B37] Hagg GM, Anderson PA, Hobart DJ and Danoff JV (1991). Static Work Loads and Occupational Myalgia - A New Explanational Model.. Electromyographical kinesiology.

[B38] Hagg GM, Luttmann A, Jager M (2000). Methodologies for evaluating electromyographic field data in ergonomics. J Electromyogr Kinesiol.

[B39] Roman-Liu D, Tokarski T, Wojcik K (2004). Quantitative assessment of upper limb muscle fatigue depending on the conditions of repetitive task load. J Electromyogr Kinesiol.

[B40] Szeto GP, Straker LM, O'Sullivan PB (2005). EMG median frequency changes in the neck-shoulder stabilizers of symptomatic office workers when challenged by different physical stressors. J Electromyogr Kinesiol.

[B41] Peper E, al (2003). The Integration of electromyography (SEMG) at the workstation: assessment, treatment, and prevention of repetitive strain injury (RSI).. Appl Psychophysiol Biofeedback.

[B42] Ware JE, Sherbourne CD (1992). The MOS 36-item short-form health survey (SF-36). I. Conceptual framework and item selection. Med Care.

[B43] Geisser ME, Roth RS, Robinson ME (1997). Assessing depression among persons with chronic pain using the Center for Epidemiological Studies-Depression Scale and the Beck Depression Inventory: a comparative analysis. Clin J Pain.

[B44] Solway S, Beaton DE, McConnell S, Bombardies C (2002). The DASH Outcome Measure User's Manual.

[B45] Turchin DC, Beaton DE, Richards RR (1998). Validity of observer-based aggregate scoring systems as descriptors of elbow pain, function, and disability. J Bone Joint Surg Am.

[B46] Beaton DE, Katz JN, Fossel AH, Wright JG, Tarasuk V, Bombardier C (2001). Measuring the whole or the parts? Validity, reliability, and responsiveness of the Disabilities of the Arm, Shoulder and Hand outcome measure in different regions of the upper extremity. J Hand Ther.

[B47] Beaton DE, Davies AM, Hudak P, McConnell S (2001). The DASH (Disabilities of the Arm, Shoulder and Hand) outcome measure: What do we know about it now?. British Journal of Hand Therapy.

[B48] Bot SD, Terwee CB, van der Windt DA, Bouter LM, Dekker J, de Vet HC (2004). Clinimetric evaluation of shoulder disability questionnaires: a systematic review of the literature. Ann Rheum Dis.

[B49] Clarkson HM (2005). Joint Motion and Function Assessment A research-based practical Guide.

[B50] A.F.de W, Heemskerk MA, Terwee CB, Jans MP, Deville W, van Schaardenburg DJ, Scholten RJ, Bouter LM (2004). Inter-observer reproducibility of measurements of range of motion in patients with shoulder pain using a digital inclinometer. BMC Musculoskelet Disord.

[B51] Gummesson C, Atroshi I, Ekdahl C (2003). The disabilities of the arm, shoulder and hand (DASH) outcome questionnaire: longitudinal construct validity and measuring self-rated health change after surgery. BMC Musculoskelet Disord.

[B52] Altman DG (1991). Practical statistics for medical research.

[B53] Thomas E, van der Windt DA, Hay EM, Smidt N, Dziedzic K, Bouter LM, Croft PR (2005). Two pragmatic trials of treatment for shoulder disorders in primary care: generalisability, course, and prognostic indicators. Ann Rheum Dis.

